# Presence of *Francisella tularensis* subsp. *holarctica* DNA in the Aquatic Environment in France

**DOI:** 10.3390/microorganisms9071398

**Published:** 2021-06-28

**Authors:** Camille D. Brunet, Aurélie Hennebique, Julien Peyroux, Isabelle Pelloux, Yvan Caspar, Max Maurin

**Affiliations:** 1 Centre National de la Recherche Scientifique, Université Grenoble Alpes, TIMC, UMR5525, 38000 Grenoble, France; camille.brunet@univ-grenoble-alpes.fr (C.D.B.); ahennebique@chu-grenoble.fr (A.H.); julien.peyroux@univ-grenoble-alpes.fr (J.P.); YCaspar@chu-grenoble.fr (Y.C.); 2 Centre National de Référence des Francisella, Centre Hospitalier Universitaire Grenoble Alpes, 38000 Grenoble, France; ipelloux@chu-grenoble.fr

**Keywords:** Tularemia, *Francisella tularensis*, zoonosis, water, France, environment and public health

## Abstract

In 2018, the incidence of tularemia increased twofold in the west of France, with many pneumonic forms, suggesting environmental sources of infection. We investigated the presence of *Francisella* *tularensis* subsp. *holarctica* and other *Francisella* species DNA in the natural aquatic environment of this geographic area. Two sampling campaigns, in July 2019 and January 2020, allowed the collection of 87 water samples. Using a combination of real-time PCR assays, we tested the presence of either *Francisella* sp., *F. tularensis/F. novicida,* and *F. tularensis* subsp. *holarctica*, the latter being the only tularemia agent in Europe. Among 57 water samples of the first campaign, 15 (26.3%) were positive for *Francisella* sp., nine (15.8%) for *F. tularensis* and/or *F. novicida*, and four (7.0%) for *F. tularensis* subsp. *holarctica*. Ratios were 25/30 (83.3%), 24/30 (80.0%), and 4/30 (13.3%) for the second campaign. Among the thirty sites sampled during the two campaigns, nine were positive both times for *Francisella* sp., seven for *F. tularensis* and/or *F. novicida*, and one for *F. tularensis* subsp. *holarctica*. Altogether, our study reveals a high prevalence of *Francisella* sp. DNA (including the tularemia agent) in the studied aquatic environment. This aquatic environment could therefore participate in the endemicity of tularemia in the west of France.

## 1. Introduction

*Francisella tularensis* is a Gram-negative bacterium that causes the zoonosis tularemia. It is a highly virulent human pathogen classified in category A of potential agents of biological threat by the US Centers for Disease Control and Prevention (CDC) [1]. Classically, four subspecies of *F. tularensis* are recognized. Only two are associated with human tularemia: *F. tularensis* subsp. *tularensis* (type A) is the most virulent, only present in North America; and *F. tularensis* subsp. *holarctica* (type B) is less virulent, and located in all the northern hemisphere and Australia [2,3]. *F. tularensis* subsp. *mediasiatica* is restricted to central Asia and Russia, and has never been associated with human infections [4]. *F. tularensis* subsp. *novicida,* is an aquatic bacterium with low virulence in humans [5] and can also be considered a different species, *F. novicida* [6,7]. Based on the most recent phylogenetic studies, we choose in the article to considered *F. novicida* as a species [8]. Other *Francisella* species are occasionally associated with human infections, like the aquatic bacterium *F. philomiragia* [5]. In the past twenty years, multiple new environmental *Francisella* species were described, all of them isolated from water or from aquatic fauna [8].

Six major clinical forms of tularemia are recognized depending on the mode of contamination. The most frequent are the ulceroglandular and glandular forms, corresponding to a regional lymphadenopathy, with or without a skin inoculation lesion. The oculoglandular and oropharyngeal forms correspond to regional lymphadenopathy with conjunctivitis or pharyngitis, respectively. Two systemic forms, which may be associated with fatality rates up to 30%, are recognized: the pneumonic form and the typhoidal form, with severe sepsis resembling typhoid [9]. The primary sources of human infections are wildlife animals (especially lagomorphs and small rodents) and arthropods (ticks, mosquitoes, and rarely biting flies) [9]. Infections through contact with a contaminated hydro-telluric environment are still poorly defined [9]. However, tularemia can also be a waterborne infection occurring after aquatic activities (e.g., fishing, swimming, and canyoning) or through drinking *F. tularensis*-contaminated water [5]. Several modes of *Francisella* survival in water are presumed: survival in a planktonic form; through interactions with amebae; in mosquito larvae; or in biofilms [5].

Several studies have reported the detection of *Francisella* sp. in environmental water samples, including in Turkey [10,11], the Netherlands [12], Germany [13], Ukraine [14], Sweden [15], Norway [16], and the USA [17,18,19,20]. To our knowledge, no similar study has been previously conducted in France.

In 2018, tularemia incidence increased twofold in France, with 21% of pneumonic cases, while this clinical form usually represents less than 10% of reported tularemia cases (data from santepubliquefrance.fr). Seventy-nine out of 113 patients with a known address lived in the west of France, especially in Pays de la Loire, Bretagne, and the north of Nouvelle Aquitaine. These infections were sporadic and spread over 65,000 km^2^ areas. Although most of the patients were farmers, their modes of infection were not identified (data from santepubliquefrance.fr). In 2019 and 2020, we carried out a study on the presence of *F. tularensis* DNA in natural aquatic environments located in the Pays de la Loire region. Our goal was to demonstrate that surface waters could represent a reservoir of this bacterium in France and a potential source of human infections.

## 2. Materials and Methods

### 2.1. Sample Collection and DNA Extraction

Environmental water samples (2 to 4 L) were collected in the Pays de la Loire region in natural aquatic sites in the areas where most tularemia cases had occurred. Fifty-seven water samples were collected in July 2019 (Figure S1). A second field study was carried out in the same geographic areas in January 2020, during which 30 water samples were collected, including 12 from previously *Francisella* PCR-positive sites, and 18 from negative sites (Figure S2). A wide diversity of water sites was sampled: ocean, rivulets, rivers, canals, ponds, lakes, and dams (see results for more details). For each sampling site, the GPS coordinates, temperature, and salinity of the collected water samples were recorded. These samples were transferred to our laboratory within two days and stored at 4 °C before testing. For PCR experiments, filtration of one liter of each water sample was performed using 0.22 µm or 0.45 µm pore size filters (ThermoFisher Scientific, Waltham, MA, USA) depending on turbidity. Total DNA was then extracted from the filters using the NucloMag DNA/RNA water kit (Macherey-Nagel, Hoerdt, France).

### 2.2. Francisella Species Detection

To identify *Francisella*-positive samples and characterize the species or subspecies present, we used three real-time PCR (qPCR) assays, employed for *Francisella* identification or tularemia diagnosis. These assays allowed detection of respectively *Francisella* sp. (ISFtu2-qPCR); *F. tularensis* (i.e., *F. tularensis* subsp. *tularensis*, subsp. *holarctica,* and subsp. *mediasiatica*) and *F. novicida* (Tul4-qPCR); and *F. tularensis* subsp. *holarctica* (Type B-qPCR) (Table 1). Each PCR mixture (20 µL) contained 10 µL of TaqMan Fast Advanced PCR Master Mix kit (ThermoFisher Scientific, Waltham, MA, USA), 0.4 µL of each 10 µM primers, 0.4 µL of 2 µM probe, 3.8 µL of water, and 5 µL of 10 ng/µL diluted DNA. The thermocycling program was the same for the three qPCR tests: 50 °C for 2 min, 95 °C for 2 min, 45 cycles at 95 °C for 3 s, and 60 °C for 30 s, using the Ligthcycler 480 (Roche Diagnostics, Meylan, France). All qPCR tests were performed in duplicate. The qPCRs were considered positive for a specific sample if the two duplicate tests were positive, with a cycle threshold (C_t_) value ≤36 for ISFtu2-qPCR, and <40 for Tul4- and Type B-qPCR tests. In the case of inconsistent results within the duplicate for a sample, this sample was tested a third time to conclude Controls were carried out using DNA extracts from strains (*Serratia marcescens* CIP 103551, *Streptococcus equi* ATCC 43079, *F. philomiragia* ATCC 25015, *F. noatunensis* LMG 23800, *F. novicida* U112, clinical strain Ft92 of *F. tularensis* subsp. *holarctica*), and with DNA extracts from *F. tularensis* subsp. *holarctica* LVS NCTC 10857-spiked water samples at concentrations ranging from 0.1 to 10,000 CFU/l (Table 2). Our *F. tularensis* collection has been approved by the Agence Nationale de Sécurité du Médicament et des Produits de santé (France) (ANSM, authorization number ADE-103892019-7).

### 2.3. Amebae Detection

*Acanthamoeba* sp. DNA was detected using a qPCR (Acanth-qPCR) targeting a 180 bp fragment of the 18 S rDNA, as previously described [21] (Table 1). Each PCR mixture (20 µL) contained 10 µL of TaqMan Fast Advanced PCR Master Mix kit (ThermoFisher Scientific, Waltham, MA, USA), 0.4 µL of each 10 µM primers, 0.4 µL of 2µM probe, 3.8 µL of water, and 5 µL of 10 ng/µL diluted DNA. The qPCR settings were as follows: 50 °C for 2 min, 95 °C for 2 min, followed by 45 cycles of 95 °C for 15 s, 55 °C for 30 s, and 60 °C for 30 s, on a LightCycler 480 (Roche Diagnostics, Meylan, France). Each water sample was tested in duplicate or triplicate for inconsistent results. Water samples were considered positive for *Acanthamoeba* sp. if the two duplicate tests were positive with a C_t_ value <40.

### 2.4. Statistical Analysis

Mann–Whitney and Khi2 tests were used to compare the influence of temperature and the presence of *Acanthamoeba* sp. on *Francisella* sp. detection, using a significance level of 0.05. Statistical analyses were not carried out on the second campaign because the sampling sites were chosen according to the first campaign results, introducing a bias.

## 3. Results

### 3.1. qPCR Controls

Regarding the bacterial strains’ controls, the ISFtu2-qPCR gave a strongly positive signal for *F. tularensis* subsp. *holarctica* and *F. novicida*. A weaker signal (C_t_ of 36) was obtained for *F. noatunensis* and *F. philomiragia*. However, non-*Francisella* strains tested also gave a weak amplification signal (C_t_ > 36) that was abolished when testing lower DNA concentrations (data not shown). Indeed, we choose to consider ISFtu2-qPCR as positive when C_t_ was inferior or equal to 36. Tul4-qPCR gave a strong positive signal for *F. tularensis* subsp. *holarctica* and *F. novicida* and was negative for all the other strains tested. Finally, Type B-qPCR was only positive for *F. tularensis* subsp. *holarctica.*

Regarding the *F. tularensis* subsp. *holarctica* LVS NCTC 10857-spiked water samples, when applying our defined thresholds, the sensitivity of our qPCR-assay was of 1 CFU/l for ISFtu2-qPCR, 10 CFU/l for Tul4-qPCR and 100 CFU/l for Type B-qPCR. The qPCR controls results are presented in Table 2.

### 3.2. First Field Study

**Figure 1:**
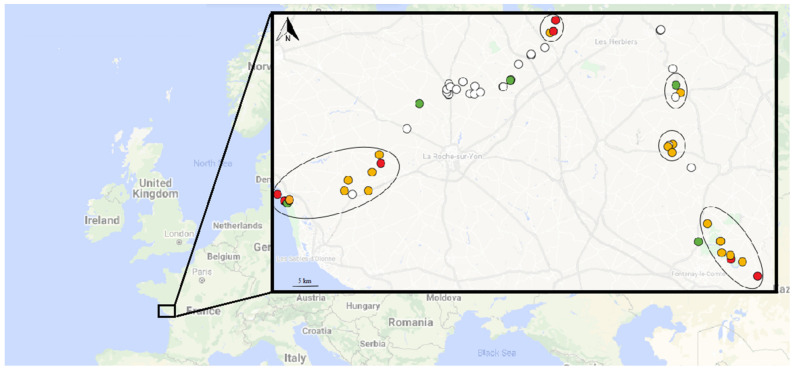
*Francisella* sp. detection in surface water samples collected during both campaigns. For each site, DNA was extracted from water, and three q-PCR tests were performed: ISFtu2-qPCR for *Francisella* sp., Tul4-qPCR for *F. tularensis/F. novicida*, and Type B-qPCR for *F. tularensis* subsp. *holarctica*. Circle: water sampling site. White circle: sample with no positive qPCR. Green circle: sample with positive ISFtu2-qPCR in the first campaign, or the second campaign, or both, and negative Tul4 and Type B-qPCR. Orange circle: sample with positive ISFtu2 and Tul4-qPCR in the first campaign, or the second campaign, or both, and negative Type B-qPCR. Red circle: sample with positive ISFtu2, Tul4, and Type B-qPCR in the first campaign, or the second campaign, or both. Disks indicate sites where water samples were collected during the two campaigns.

Regarding the first field study, 57 water samples were collected: one from the ocean, two from canals near the ocean, one from a river near the ocean, one from a pond near the ocean, 30 from ponds, 12 from rivers (some of them were sampled at several points), two from rivulets, three from lakes, three from dams, and two from canals. Fifteen (26.3%) out of 57 collected water samples were positive for ISFtu2-qPCR, indicating the presence of *Francisella* sp. at the corresponding sampling sites. Among the 15 samples positive for ISFtu2-qPCR, nine were also positive for Tul4-qPCR, indicating the presence of *F. tularensis,* or *F. novicida*, or both. Four samples were positive for the three qPCR tests, including Type B-qPCR, confirming the presence of *F. tularensis* subsp. *holarctica* (Figure 1 and Figure S1). One river was found contaminated at two sampling sites. Characteristics of the positive water samples are presented in Table S1. None of the ISFtu2-qPCR negative samples gave an amplification signal with the Tul4- or Type B-qPCR tests.

Salinity was evaluated for 12 of the 15 ISFtu2-qPCR-positive samples and ranged from 1 g/L to 38 g/L. Indeed, the ISFtu2-qPCR-positive samples corresponded either to saltwater (three samples), brackish water (one sample), or freshwater (eight samples). Salinity was evaluated for 19 of the 42 ISFtu2-qPCR-negative samples and ranged from 1 g/L to 46 g/L. The impact of salinity on *Francisella* sp. detection could not be evaluated due to too few salt- and brackish water samples. The water temperature of the 15 ISFtu2-qPCR-positive samples ranged from 18.2 °C to 27.8 °C, and that of the 42 ISFtu2-qPCR-negative samples from 16.2 °C to 29.9 °C. There was no significant difference between the average temperature of ISFtu2-qPCR-positive and -negative samples (*p* = 0.809). *Acanthamoeba* sp. DNA was detected in 38 (67.9%) of the 56 tested surface water samples. For these samples, seven (18.4%) were positive for ISFtu2-qPCR, and 31 (81.6%) were negative. For the 18 *Acanthamoeba*-negative samples, seven (38.9%) were positive for ISFtu2-qPCR, and 11 (61.1%) were negative. There was no significant correlation between the distribution of positive or negative samples for ISFtu2-qPCR and *Acanthamoeba*’s presence or absence (*p* = 0.063).

### 3.3. Second Field Study

Regarding the second field study that was conducted mainly around the locations that were positives during the first field study, the ISFtu2-qPCR was positive for 25/30 (83.3%) samples, the Tul4-qPCR for 24/30 (80.0%), and the Type B-qPCR for 4/30 (13.3%) (Figure 1 and Figure S2). Two rivers were found contaminated at three or more sampling sites. Here again, none of the ISFtu2-qPCR negative samples gave an amplification signal with the Tul4- or Type B-qPCR tests. The salinity ranged from 1 g/L to 12 g/L for the 25 ISFtu2-qPCR-positive samples, and 1 g/L to 31 g/L for the five ISFtu2-qPCR-negative samples. The temperature ranged from 7.0 °C to 13.6 °C for the 25 ISFtu2-qPCR-positive samples and 6.6 °C to 13.2 °C for the five ISFtu2-qPCR-negative samples. Finally, 29/30 (96.7%) water samples collected during this second campaign were positive for *Acanthamoeba* sp. PCR.

Of the 12 sites that were ISFtu2-qPCR positive during the first water sampling campaign, and re-sampled during the second campaign, nine were still positive for this qPCR test, suggesting the persistence of *Francisella* sp. DNA over time, or a regular recontamination of the environment, or both. For the Tul4-qPCR, seven of the nine previously positive sites were still positive during the second campaign. Finally, of the four sites determined as Type B-qPCR positive during the first water sampling campaign, one remained positive.

Furthermore, among the 18 water samples that were negative for the ISFtu2-qPCR test during the first campaign, 16 tested positive for this PCRduring the second campaign. Of these 16 samples, 15 also tested positive for Tul4-qPCR, and two for both Tul4- and Type B-qPCRs. Characteristics of the positive water samples are presented in Table S1.

## 4. Discussion

A significant increase in tularemia cases was experienced in 2018 in France. A doubling of the disease incidence was observed in the western part of the country compared to previous years. Because of the rise in the pneumonic forms of the disease (21% of cases in 2018 vs. 10% in previous years), inhalation of *F. tularensis*-contaminated aerosols could be suspected as one of the significant sources of human infections. As these pneumonic tularemia cases occurred throughout the whole year, the long-term environmental reservoir of this bacterium was hypothesized.

This study aimed to assess the prevalence and the spatial and temporal distribution of *F. tularensis* in surface waters. For this purpose, two sampling campaigns were performed six months apart, in July 2019 and January 2020, to collect natural environmental water sources in the geographic area where most tularemia cases occurred.

A dozen studies have evaluated the presence of *Francisella* sp. in aquatic environments [10,11,12,13,14,15,16,17,18,19,20]. The previous studies on environmental water samples mainly detected *F. philomiragia* or *F. novicida* [16,17,18,19,20], which are well-known aquatic bacteria [5]. Few environmental studies identified *F. tularensis* in water samples [10,11,12,13,14,15]. Most of these previous studies performed on environmental water samples relied on PCR assays [10,11,12,13,15,16,17,19], while few studies used culture methods [10,14,18,19,20]. Isolation of *F. tularensis* from environmental water samples has been only rarely reported [10,14]. Environmental surface water samples are contaminated by a wide diversity of bacteria that grow faster than the fastidious *Francisella*, thus preventing the latter’s isolation. Consequently, we did not try to culture our water samples, although it would have been interesting to correlate the PCR and culture results. PCR-based methods seem more suitable for detecting *Francisella* in environmental samples. However, a wide diversity of *Francisella* species is present in the environment, including many water species (e.g., *F. ulginis*, *F. salminarina*, *F. salina*), fish- and mollusc-associated species (e.g., *F. noatunensis*, *F. orientalis*, *F. halioticida*, *F. marina*), and endocytobionts of marine ciliates (e.g., *F. endociliophora* and *F. adeliensis*) [8]. Consequently, the choice of PCR targets is critical for the nature of *Francisella* species identified. Thus, we performed a set of three real-time PCR tests allowing us to discriminate between *Francisella* species and subspecies. The ISFtu2-qPCR assay targets the insertion element-like sequence IS*Ftu2*, which is present in multiple copies in the *F. tularensis* (i.e., *F. tularensis* subsp.* tularensis*, subsp. *holarctica* and subsp. *mediastitica*) and the *F. novicida* genome (i.e., there are 12–17 copies for subsp. *tularensis*, 26–30 copies for subsp. *holarctica*, and 6–18 copies for *F. novicida*) and thus improves the sensibility of the qPCR. This DNA target is present in other *Francisella* species (i.e., there are 1–2 copies of IS*Ftu2* in the *F. philomiragia* genome) [8,22]. Consequently, qPCR results from the PCR targeting the IS*Ftu2* sequence should be interpreted cautiously because it could detect a wide diversity of *Francisella* species. This was observed with our ISFtu2-qPCR controls, which demonstrated both detection of *F. philomiragia* and *F. noatunensis*. Öhrman et al., by in silico approaches, demonstrated that ISFtu2-qPCR can detect *F. novicida* and *F. philomiragia* but does not detect other *Francisella* species such as *F. opportunistica* and *Francisella* endocytobionts [8]. Consequently, the ISFtu2-qPCR is a *Francisella* sp. PCR with the limitation that not all the described *Francisella* species are detected. The Tul4-qPCR assay targets a simple copy gene, which can be found in *F. tularensis* and in *F. novicida* [22]. Although our Tul4-qPCR controls and those of Versage et al. [22] concluded that *F. philomiragia* and other *Francisella* near neighbors were not amplified by this assay, Öhrman et al., by in silico approaches found that *F. philomirgia* can occasionally be amplified by the Tul4-qPCR [8]. Finally, the Type B-qPCR test targets a specific junction between IS*Ftu2* and a flanking 3’ region, which is only found in *F. tularensis* subsp. *holarctica* [23], the only tularemia agent found in Europe [9]. Thus, our multi-step PCR approach, using ISFtu2-, Tul4- and Type B-qPCR, allowed reliable detection of respectively *Francisella* sp., *F. tularensis*/*F. novicida*, and *F. tularensis* subsp. *holarctica*.

Regarding the first collection campaign, 26.3% of samples were positive only for ISFtu2-qPCR, 15.8% for ISFtu2- and Tul4-qPCR, and 7.0% for ISFtu2-, Tul4-, and Type B-qPCR. To be as stringent as possible, these samples were classified as containing *Francisella* sp., *F. tularensis* and/or *F. novicida*, or *F. tularensis* subsp. *holarctica*, respectively. Because the qPCR targeting the multi-copy IS*Ftu2* sequence is more sensitive than the two other qPCR tests, samples with only a positive ISFtu2-qPCR could also correspond to *F. tularensis* or even *F. tularensis* subsp.* holarctica*. Therefore, a 7% prevalence of water samples contaminated with *F. tularensis* subsp. *holarctica* should be considered a minimum in this study. The effect of salinity on *Francisella* sp. detection could not be evaluated because of too few salt- and brackish water samples being included. However, *Francisella* sp. were detected in fresh-, brackish-, and saltwater, indicating that these bacteria could contaminate a wide water-type diversity. Similarly, in the Netherland, Janse et al. identified *Francisella* sp. in fresh-, brackish- and saltwater samples [12]. Interestingly, in Norway, Duodu et al. found *Francisella* sp. in 44 seawater samples, while all 128 freshwater samples were free of *Francisella* sp. [16]. In our study, no significant difference in water temperature was observed between *Francisella* sp.-positive and -negative samples. In Norway, Duodu et al. also observed that water temperature had no effect on *Francisella* sp. presence in water, but they only found *Francisella* sp.-positive samples south of the Arctic Circle, suggesting that climate could have an impact on *Francisella* sp. presence in the aquatic environment [16].

The second collection campaign aimed to assess the possible persistence of *Francisella* sp. in previously investigated aquatic sites. Therefore, 30 of the previously collected sites were resampled, including 12 that were positive for *Francisella* sp. and 18 that were negative but located close to positive sampling points. Among the 12 samples positive for ISFtu2-qPCR during the first campaign, nine (75.0%) were still positive during the second campaign. As for the Tul4- and Type B-qPCR, 7/9 and 1/4 of the previously positive sites were still positive during the second campaign. Thus, one water sample was positive for the tularemia agent *F. tularensis* subsp. *holarctica* both in July 2019 and in January 2020. These results indicate either the persistence of *Francisella* sp. in the corresponding surface-water sites for several months, or repeated contamination over time of these aquatic environments, or both. Each of these situations corresponds to a high risk of occurrence of human contamination from these aquatic reservoirs. Interestingly, 16/18 (88.9%) of the previously *Francisella* sp.-negative sites were ISFtu2-qPCR-positive during the second campaign, arguing that *Francisella* sp. contamination of the aquatic environment can be variable in time and space, and might spread over time. These observed variations impel that repeated sampling is required for *Francisella* environmental monitoring. Overall, these results highlight that *Francisella* sp. contamination of the water environment is recurrent whatever the season.

In total, there was respectively 26.3% and 83.3% of ISFtu2-qPCR-positive samples in the first collection campaign and the second collection campaign. These percentages of ISFtu2-qPCR-positive samples are high and indicate that *Francisella* sp. contamination of the environment is widespread. Janse et al. previously described in the Netherlands that 88% of water samples were positive for *Francisella* sp. in areas with reported tularemia cases, and 10% in randomly collected sites [12]. Broman et al., in Sweden, collected 341 fresh surface water samples during three years and found 108 (32%) samples positives for *F. tularensis* [15]. In contrast, other studies reported a lower prevalence of *Francisella* sp. in the environment. Kaysser et al. in Germany sampled 28 freshwater sites and found one (4%) PCR-positive sample [13]. Simşek et al. collected 154 water samples from rivers, spring water, and village fountains after tularemia outbreaks in Turkey, and detected *F. tularensis* subsp. *holarctica* by culture in four samples and by PCR in 17 (11%) samples [10]. However, it would have been interesting to include in this study samples from a control location with lower tularemia incidence in order to compare frequency of contamination by *F. tularensis* DNA.

Altogether, our study in the West of France demonstrates that the presence of *F. tularensis* subsp. *holarctica* DNA in surface waters is frequent and may be recurrent in areas close to human tularemia cases. Although the presence of *F. tularensis* DNA in surface water samples is not evidence of human contamination, the presence of this bacterium over a wide range of time and space could favour the occurrence of human infections in the corresponding geographic area. Indeed, the high number of pneumonic tularemia cases in 2018 suggested an environmental mode of contamination. Although aerosol inhalation from contaminated soil is considered the primary source of pneumonic tularemia [24,25], we hypothesis that the persistence of *F. tularensis* in the aquatic environment could explain some of these tularemia cases [5]. Waterborne tularemia cases classically occur through drinking contaminated water or direct contact with contaminated water sources, especially during recreational activities such as fishing and swimming [5]. However, contact or consumption of contaminated water usually leads to skin or oropharyngeal inoculation of *F. tularensis*; thus, ulceroglandular or oropharyngeal forms of tularemia emerge. We hypothesize another water-related human contamination leading to the disease’s pulmonary form through aerosols formed from contaminated surface water. Indeed, the 2018 tularemia cases occurred in areas with extensive agricultural activities, and most patients were farmers. We noted that most of the crop fields of the studied areas were irrigated by sprinklers using surface waters, which could generate large aerosols.

The widespread detection of the tularemia agent *F. tularensis* subsp. *holarctica* in surface water raises the question of how this bacterium can survive and persist in such aquatic environments and why it does not infect thousands of people, according to the high virulence of this bacterium. Experimental studies have shown that *F. tularensis* can survive in water microcosms for months [26,27,28,29], firstly in a culturable form and then in a potentially viable but nonculturable form (VBNC) [26,28]. Long-term planktonic forms of *F. tularensis* subsp. *holarctica* have been found to be still virulent in mice, whereas similar forms of *F. tularensis* subsp. *tularensis* did not [29]. VBNC forms of *F. tularensis* subsp. *holarctica* appeared to be no longer virulent in mice [28]. This possible loss of virulence over time in aquatic environments could explain the relatively low number of tularemia cases related to aquatic habitats in endemic areas compared to the virulence of the bacteria in its virulent state. In our study, the DNA detection of the tularemia agent does not allow us to determine the detected bacteria’s infectivity. Several in vitro experimental studies have shown that *F. tularensis* can survive in water through interaction with amebae [30,31,32,33]. *Acanthamoeba* species were especially shown to promote *Francisella* sp. survival in water and are ubiquitous environmental amebae [34] that is why we researched DNA from *Acanthamoeba* in our water samples. We detected DNA from *Acanthamoeba* sp. in 38/56 (67.9%) of the water samples of the first collection campaign, and 29/30 (96.7%) of the water samples of the second collection campaign. However, no correlation was found between the presence of *Acanthamoeba* and *Francisella* DNA in water samples. There are conflicting studies about the capacity of *F. tularensis* to produce biofilms to survive in water [29,35]. Finally, this bacterium was shown to be able to survive within mosquito larvae [36]. While the tularemia agent aquatic cycle is not yet fully characterized, our work demonstrates that *F. tularensis* subsp. *holarctica* DNA can be found in the aquatic environment.

In conclusion, our study demonstrates, for the first time, the presence of *F. tularensis* subsp. *holarctica* DNA in surface water in France. Although contaminated water as a source of pneumonic forms of human tularemia remains to be firmly established, the monitoring of *F. tularensis* contamination of surface water should be considered for public health surveillance of tularemia.

## Figures and Tables

**Table 1:** Primers and probes used for *Francisella* sp. and *Acanthamoeba* sp. detection.

qPCR (Target)	Primers/Probes	5′ to 3′ DNA Sequence	Amplicon Size	Species Detected	Reference
ISFtu2-qPCR (IS*Ftu2* insertion sequence element)	Forward	ttggtagatcagttggtgggataac	97 bp	*Francisella* sp.	[22]
	Reverse	tgagttttaccttctgacaacaatatttc			
	Probe	FAM-aaatccatgctatgactgatgctttaggtaatcca-BHQ1			
Tul4-qPCR (*tul4* gene)	Forward	attacaatggcaggctccaga	91 bp	*F. tularensis* and *F. novicida*	[22]
	Reverse	tgcccaagttttatcgttcttct			
	Probe	FAM-ttctaagtgccatgatacaagcttcccaattactaag-BHQ1			
Type B-qPCR (junction between IS*Ftu2* and a specific flanking 3′ region)	Forward	cttgtacttttatttggctactgagaaact	144 bp	*F. tularensis* subsp. *holarctica*	[23]
	Reverse	cttgcttggtttgtaaatatagtggaa			
	Probe	FAM-acctagttcaacc*t*caagacttttagtaatgggaatgtca-BHQ1 Internal quencher			
Acanth-qPCR (*Acanthamoeba* sp. 18 S rDNA)	Forward	cccagatcgtttaccgtgaa	180 bp	*Acanthamoeba* sp.	[21]
	Reverse	taaatattaatgcccccaactatcc			
	Probe	FAM-ctgccaccgaatacattagcatgg-BHQ1			

**Table 2:** The qPCR control results.

DNA Extracts	ISFtu2-qPCR (C_t_)	Tul4-qPCR (C_t_)	Type B-qPCR (C_t_)
*Serratia marcescens* CIP 103551 *	37	Negative	Negative
*Streptococcus equi* ATCC 43079 *	37	Negative	Negative
*F. noatunensis* LMG 23800 *	36	Negative	Negative
*F. philomiragia* ATCC 25015 *	36	Negative	Negative
*F. novicida* U112 *	12	16	Negative
Clinical strain Ft92 of *F. tularensis* subsp. holarctica *	12	15	16
LVS-spiked water 0.1 CFU/l	38	Negative	Negative
LVS-spiked water 1 CFU/l	36	Negative	Negative
LVS-spiked water 10 CFU/l	36	37	Negative
LVS-spiked water 100 CFU/l	34	38	38
LVS-spiked water 1000 CFU/l	27	33	33
LVS-spiked water 10,000 CFU/l	25	30	30

* 50 ng of DNA introduced in the PCR mix.

## Supplementary Materials

The following are available online at https://www.mdpi.com/article/10.3390/microorganisms9071398/s1, Figure S1: *Francisella* sp. detection in surface water samples collected during July 2019; Figure S2: *Francisella* sp. detection in surface water samples collected during January 2020; Table S1: Characteristics of *Francisella* sp.-positive water samples.
