# Supplementary material for: Presence of Francisella tularensis subsp. holarctica DNA in the Aquatic Environment in France
Source: Microorganisms. 2021 Jun 28;9(7):1398. doi: 10.3390/microorganisms9071398 (PMC8306966; doi:10.3390/microorganisms9071398)
Supplement: Supplementary file 1 [file microorganisms-09-01398-s001.zip › microorganisms-1272639-supplementary.pdf]

microorganisms

MDPI

Supplementary Materials

Table S1. Characteristics of *Francisella* sp. positive water samples.

Site of water sampling	Type of water sample	First campaign						Second campaign					
		T °C	NaCl (g/L)	ISFtu2-qPCR (average Ct)	Tul4-qPCR (average Ct)	Type B-qPCR (average Ct)	Acant-qPCR	T °C	NaCl (g/L)	ISFtu2-qPCR (average Ct)	Tul4-qPCR (average Ct)	Type B-qPCR (average Ct)	Acant-qPCR
**J1P1**	Ocean	21.0	37	+ (31.1)	+ (36.5)	+ (35.8)	+	11.0	31				+
**J1P2**	Canal near ocean	25.6	16	+ (32.7)	+ (36.4)	+ (36.9)		11.6	2				+
**J1P3**	Canal near ocean	27.2	38	+ (35.6)				13.2	3				+
**J1P4**	River near ocean	23.8	37	+ (32.1)	+ (36.8)		NP	7.0	6	+ (35.4)	+ (37.3)		+
**J1P5**	Pond near ocean	27.8	46					13.6	12	+ (33.2)	+ (34.4)		+
**J1P6**	Pond	27.1	2					12.6	2	+ (34.9)	+ (37.6)		+
**J1P7**	Pond	29.9	1					12.2	3	+ (31.8)	+ (35.9)		+
**J1P9**	Pond	27.8	2	+ (36.0)	+ (37.6)			12.4	2	+ (33.3)	+ (36.6)		+
**J1P11**	Pond	28.8	1					9.2	2	+ (33.5)	+ (37.1)		
**J1P12A**	Pond	18.6	2					12.9	2	+ (32.5)	+ (35.7)	+ (38.2)	+
**J1P13**	River	18.7	2	+ (35.4)				12.7	2	+ (33.5)	+ (38.1)		+
**J2P1**	River	18.8	NP	+ (36.0)			+	NS	NS	NS	NS	NS	NS
**J2P16SB**	River	18.2	NP	+ (35.0)			+	NS	NS	NS	NS	NS	NS
**J2P22**	Pond	25.9	NP					11.8	1	+ (33.3)	+ (36.5)		+
**J2P23A**	Pond	21	2				+	13.3	2	+ (33.5)	+ (36.4)		+
**J2P23B**	River	23.7	NP				+	13.0	1	+ (30.9)	+ (34.7)	+ (39.4)	+
**J2P24**	Pond	22.3	2	+ (35.6)	+ (38.4)			13.1	2	+ (33.9)	+ (37.2)	+ (38.8)	+
**J2P42**	Pond	23.7	3				+	9.0	2	+ (34.1)			+
**J2P43**	Lake	24.4	2	+ (36.0)	+ (38.4)		+	12.3	1	+ (33.3)	+ (36.4)		+
**J2P52A**	Pond	24.7	1				+	12.0	2	+ (33.5)	+ (36.4)		+
**J2P53SA**	Pond	23.2	2					10.6	2	+ (35.1)	+ (37.7)		+

**J2P54A**	Pond	24.4	1				+	11.6	1	+ (32.4)	+ (35.9)		+
**J2P55SA**	Pond	20.0	NP	+ (35.7)			+	NS	NS	NS	NS	NS	NS
**J3P1**	Dam on river*	20.3	2	+ (32.8)	+ (37.0)			11.3	2	+ (32.7)	+ (37.4)		+
**J3P6SA**	River*	25.6	1	+ (34.5)				12.3	2	+ (33.6)	+ (37.8)		+
**J3P6SB**	River*	21.6	2				+	12.3	2	+ (34.5)	+ (37.2)		+
**J3P7**	River£	21.6	1				+	10.6	1	+ (35.1)	+ (37.3)		+
**J3P8**	Dam£	25.6	1	+ (32.0)	+ (35.5)	+ (37.4)	+	13.0	2	+ (35.1)	+ (38.4)		+
**J3P8SA**	River£	20.1	1					11.6	2	+ (34.6)	+ (37.1)		+
**J3P9**	River£	23.3	2				+	12.3	2	+ (34.5)	+ (36.5)		+
**J3P9SA**	River	19.7	2	+ (31.9)	+ (37.5)	+ (36.5)	+	13.0	2	+ (32.9)	+ (34.9)	+ (37.6)	+

For ISFtu2-qPCR : + = positive in duplicate with Ct ≤ 36 ; For Tul4-qPCR, Type B-qPCR and Acanth-qPCR : + = positive in duplicate ; NP = not performed ; NP = not performed ; NS = not sampled in the second campaign ; * and £: samples from the same river .

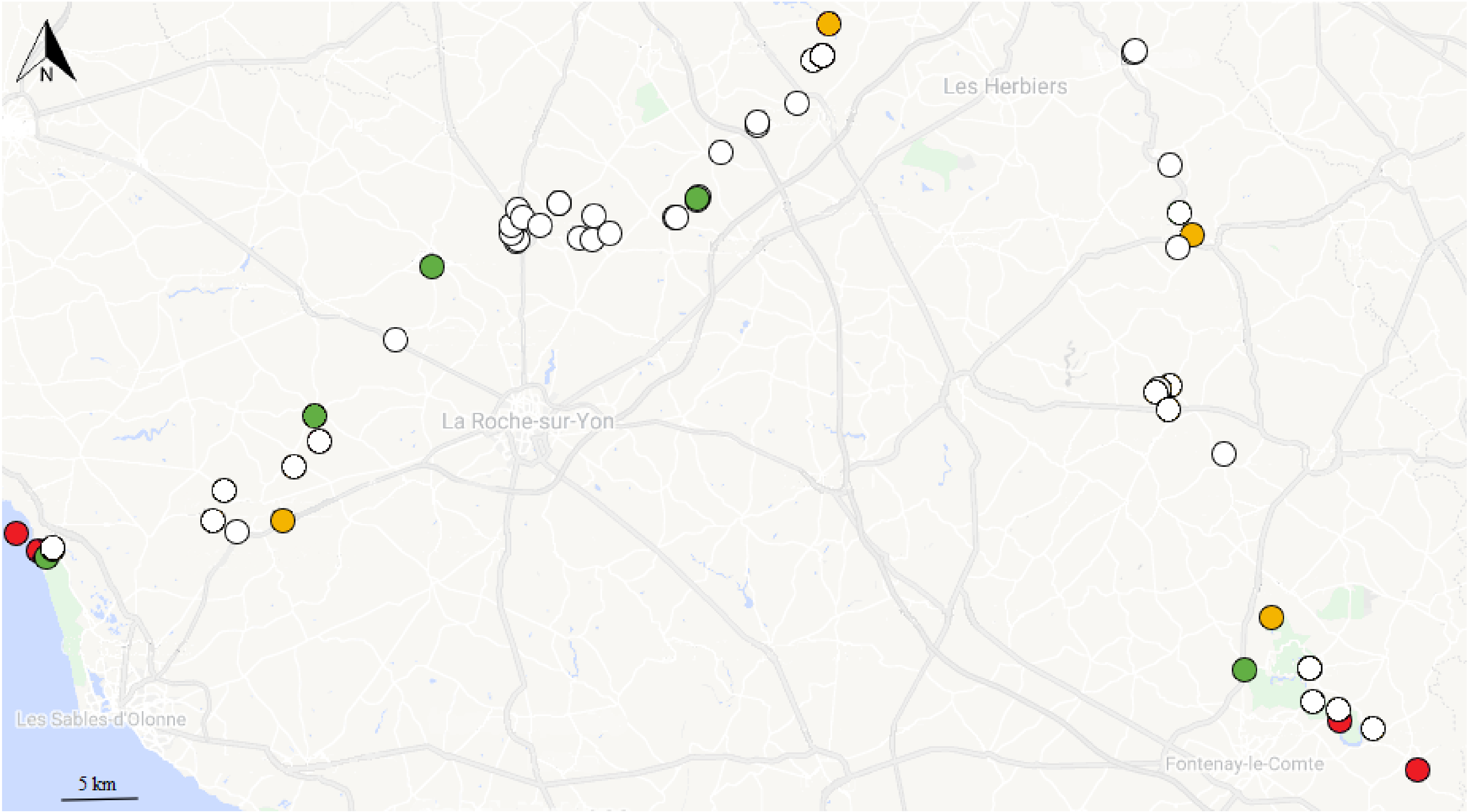

Figure S1. Francisella sp. detection in surface water samples collected during July 2019. For each site DNA were extracted from water and three q-PCR were performed (ISFtu2-qPCR for *Francisella* sp., Tul4-qPCR for *F. tularensis*, and Type B-qPCR for *F. tularensis* subsp. *holarctica*). White disk: sample with no positive qPCR. Green disk: sample positive for ISFtu2-qPCR and negative for Tul4 and Type B-qPCR. Orange disk: sample positive for ISFtu2 and Tul4-qPCR and negative for Type B-qPCR. Red disk: sample positive for the three qPCR tests.

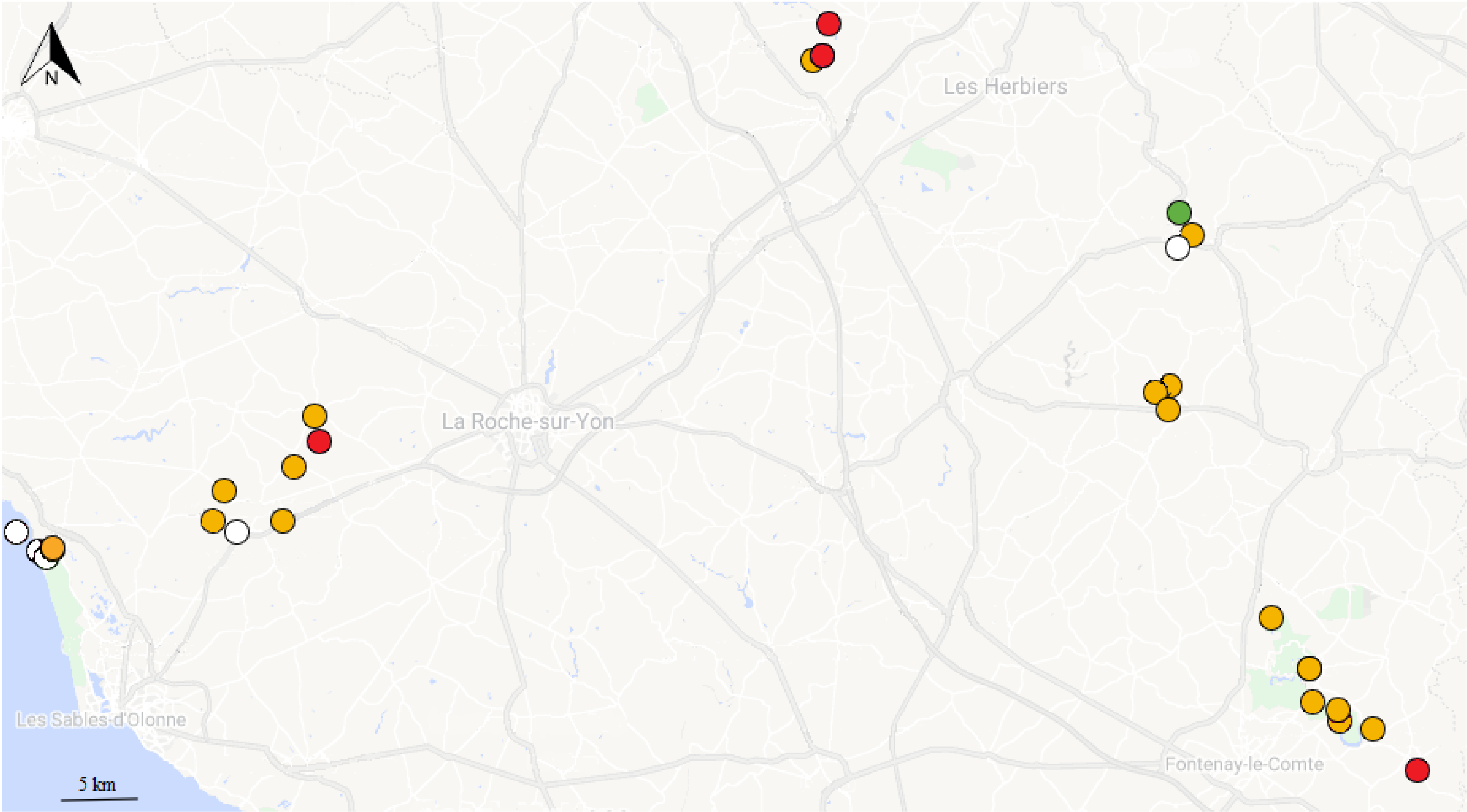

Figure S2. Francisella sp. detection in surface water samples collected during January 2020. For each site DNA were extracted from water and three q-PCR were performed (ISFtu2-qPCR for *Francisella* sp., Tul4-qPCR for *F. tularensis*, and Type B-qPCR for *F. tularensis* subsp. *holarctica*). White disk: sample with no positive qPCR. Green disk: sample positive for ISFtu2-qPCR and negative for Tul4 and Type B-qPCR. Orange disk: sample positive for ISFtu2 and Tul4-qPCR and negative for Type B-qPCR. Red disk: sample positive for the three qPCR tests.
